# Noise Reduction Characteristics of Macroporous Asphalt Pavement Based on A Weighted Sound Pressure Level Sensor

**DOI:** 10.3390/ma14164356

**Published:** 2021-08-04

**Authors:** Feng Lai, Zhiyong Huang, Feng Guo

**Affiliations:** 1 School of Highway, Chang’an University, Middle Section of South Second Ring Road, Xi’an 710064, China; 2017021068@chd.edu.cn; 2 School of Civil Engineering and Transportation, South China University of Technology, Wushan Road, Tianhe District, Guangzhou 510641, China; 3 Department of Civil and Environmental Engineering, University of South Carolina, Columbia, SC 29201, USA; fengg@email.sc.edu

**Keywords:** asphalt pavement, accelerated loading test, weighted sound pressure level, pavement noise, noise spectrum

## Abstract

Based on the manual of macroporous noise-reducing asphalt pavement design, the indoor main drive pavement function accelerated loading test system was applied to investigate the impact of speed, loading conditions (dry and wet) and structural depth on the noise reduction of macroporous Open Graded Friction Course (OGFC) pavement, as well as its long-term noise reduction. Combined with the noise spectrum of the weighted sound pressure level, the main components and sensitive frequency bands of pavement noise under different factors were analyzed and compared. According to experimental results, the noise reduction effect of different asphalt pavements from strong to weak is as follows: OGFC-13 > SMA-13 > AC-13 > MS-III. The noise reduction effect of OGFC concentrates on the frequency of 1–4 kHz when high porosity effectively reduces the air pump effect. As the effect of wheels increases and the depth of the road structure decreases, the noise reduction effect of OGFC decreases. It indicates the noise reduction performance attenuates at a later stage, similar to the noise level of densely graded roads.

## 1. Introduction

With the economic development, the number of vehicles has increased rapidly. Concomitant with this, the traffic noise pollution caused by vehicles has become more and more serious, not only bringing negative impacts on the surrounding ecological environment but also endangering human health [1,2,3,4]. Traffic noise mainly includes vehicle noise and pavement noise, and the latter is caused by the friction between tires and the road during acceleration, deceleration and braking of the vehicle, which is greatly affected by traffic conditions, road type, road gradient, driving speed, etc. [5,6]. The shaping factors of pavement noise are air pumping effect, tire vibration and aerodynamic noise [7]. According to previous studies, when the speed of a car is over 30 km/h and the speed of a truck or bus is over 50 km/h, pavement noise becomes the main component of traffic noise [8].

The influence factors of tire or pavement noise can be divided into tire factors and road factors. The former factor mainly includes tire structure and tread pattern design, and the latter factor includes the surface structure characteristics of the road surface and its own acoustic impedance (or sound absorption) characteristics, etc. [9,10,11]. Asphalt pavement is designed to provide a safe [12], high performance [13,14,15,16], long service life [17,18,19,20] and comfortable driving with low road noise [4,7,8]. The road industry usually designs low-noise pavement to reduce tire pavement noise, and the representative pavement is porous asphalt pavement (OGFC asphalt pavement) [21]. According to the previous study [22,23], the noise generated by OGFC with a thickness of 40–50 mm is 3–6 dB lower than that of ordinary asphalt concrete pavement, which is equivalent to half of the traffic volume. The research and application of macroporous asphalt pavement started early in China. However, it is limited to a large-scale application due to its short service life. The connecting pores of macroporous asphalt pavement are easily blocked, leading to excessively rapid degradation of drainage and noise reduction performance. As a result, how to balance the durability of the macroporous asphalt mixture with the anti-skid and noise reduction function to optimize the design of the mixture ratio has attracted great attention.

Currently, the method of outdoor pavement noise testing is relatively mature, which mainly uses special vehicles or trailers to collect noise on the test road at different driving speeds, expressed by sound pressure levels [24,25]. To follow the design manual, it is necessary to carry out a large number of designs of noise-reducing asphalt mixtures and to perform amount-of-noise tests [26,27]. Due to limited indoor fields and the size of the test piece, it is difficult to use the vehicle driving mode to conduct tests. Chang’an University has developed an indoor test method, which simulates the noise generated by the interaction between tires and the road surface when the vehicle is running, making the test tire roll freely from the track with a certain slope and dive onto the road slab test piece. Additionally, it can analyze the noise characteristics of the moment when the tire is in contact with the road slab test piece [28]. This method greatly reduces the cost of paving the test road, but it has high requirements for background noise control, and it is difficult to control the tire rolling speed during the test [29,30].

In order to accurately and robustly test the driving noise between the tire and different pavement, this study adopts the main driving road function accelerated loading test system, which is an all-weather road function accelerated loading simulation test system and can realize the real simulation of the interaction between the tire and the road. This system can be used to control the variable parameters, such as test temperature, humidity, ultraviolet aging, rainfall, tire axle load, the number of actions, etc. The noise law and noise reduction mechanism of different asphalt pavements are comprehensively analyzed to identify the main influence factors of pavement noise. Besides, the study of the attenuation law of pavement noise reduction performance under long-term action was conducted, aiming to provide a reference for the optimization design of durable noise reduction pavement.

### 1.1. Tire Noise Generation Mechanism

**Figure 1:**
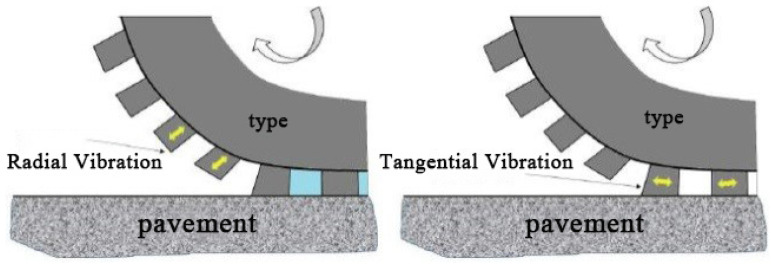
Tire vibration and noise.

Tire or pavement noise can be divided into tire vibration noise and aerodynamic noise, and the former one is caused by the impact of tread patterns on the road surface, vibration due to an uneven road surface, the friction between tires and road surface, etc. [5]. As shown in Figure 1, during the process from tire tread touching the road to leaving, the tread pattern impacts on the road surface and the deformation of tread cause the vibration along the radial direction of tread, and this induces the whole tread and sidewall vibrations. The noise pressure mainly depends on the tire rubber material, tread pattern design and road smoothness characteristics. Vibration noise is one of the sources of high-frequency part of tire or pavement noise (noise frequency is greater than 2 kHz) [7].

**Figure 2:**
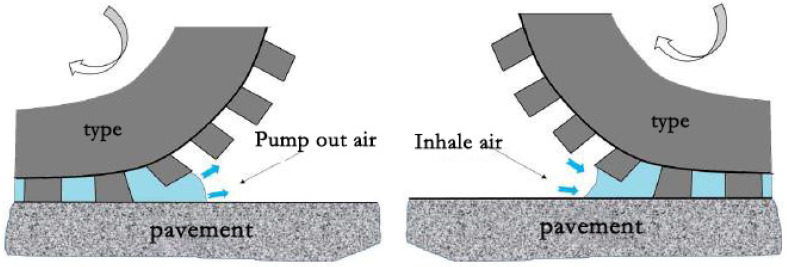
Air pumping effect.

Aerodynamic noise includes air pumping effect, air turbulence effect, acoustic tube resonance effect and Helmholtz resonance effect, among which air pumping noise contributes the most to aerodynamic noise [31]. When the tire is rolling on the pavement, once the tread of the contact part is deformed, there will be many small cavities between the tread patterns and the pavement. The tire is in close contact with the pavement during its advancement, the air in the small cavity is squeezed, part of the air is discharged to form a partial unstable air flow, and the remaining part forms a larger pressure air mass in the cavity. When the tire leaves the pavement, the volume of the compressed small cavity suddenly increases, forming a partial vacuum, and the air is sucked in abruptly. This phenomenon is called the “air pumping effect”, as shown in Figure 2. Air pumping noise frequency is around 1 kHz in the middle frequency range, which is the main source of high-speed driving noise [32].

### 1.2. Noise Evaluation Based on a Weighted Sound Pressure Level

The sound volume can be characterized by the amplitude, frequency and phase of the sound pressure, which is a function of space and time. In order to describe it more accurately, the sound pressure level is usually used to express the acoustic value. The mathematical expression is:

(1) $$\bar{L_{p}}=10\mathrm{lg}\left[\frac{1}{N}\overset{N}{\underset{i=1}{\sum }}10^{0.1\left(L_{pi}-K_{li}\right)}\right]-K_{2}-K_{3}$$

In the formula, $\bar{L_{p}}$ is the sound pressure level of the test object, dB (A); *N* is number of measuring points per unit time; $L_{pi}$ is the sound pressure level of point i, dB (A); $K_{li}$ is the background noise correction value of point I; $K_{2}$ is the correction value for the use environment; $K_{3}$ is the correction value of temperature and air pressure.

Human ears are different in sensitivity to noise of different frequencies. Therefore, in order to make the objective measurement of pavement noise consistent with human hearing, a certain frequency weighting network correction is required for pavement noise, among which the A-weighted sound pressure level frequency response is closest to the human hearing characteristics. The measurement method is simple and reasonable and has become the most widely used evaluation parameter for noise measurement. According to the international standard IEC 61672A, the A-weighting network curve function is:

(2) $$A\left(f\right)=20\mathrm{lg}\left[\frac{f_{4}^{2}f^{4}}{\left(f^{2}+f_{1}^{2}\right){\left(f^{2}+f_{2}^{2}\right)}^{\frac{1}{2}}{\left(f^{2}+f_{3}^{2}\right)}^{\frac{1}{2}}\left(f^{2}+f_{4}^{2}\right)}\right]-A_{1000}$$

In the formula, $A_{1000}$ is the sound pressure level of 1 kHz; f represents the calculation frequency; $f_{1}$ = 20.6 Hz, $f_{2}$ = 107.7 Hz, $f_{3}$ = 737.9 Hz and $f_{4}$ = 12194 Hz.

Moreover, the sound transmission has significant time and frequency domain characteristics; that is, pavement noise is essentially composed of sounds of different frequencies. In order to get the whole picture of the characteristics of tire pavement noise on different roads, it is necessary to convert the time-domain waveform of pavement noise into a spectrogram through the Fast Fourier Transform (abbreviated as FFT) algorithm. The converted frequency domain oscillogram shows the frequency composition of the noise and the corresponding signal energy. The structure is analyzed to determine the specific noise source for special noise control.

The real-time processing of sound signals is realized to ensure the speed of calculation when there are a lot of data, mainly using the FFT algorithm. FFT expression is as follows:

(3) $$X\left(f\right)=\overset{\infty }{\underset{-\infty }{\int }}x\left(t\right)e^{-2\pi ft}dt$$

In the formula, x(t) is a continuous time-domain signal of pavement noise; f is the frequency that needs to be analyzed; X(f) is the frequency-domain signal obtained after FFT of x(t). The algorithm converts the pavement noise collected in the indoor test from the time-domain signal, which is difficult to process, into the frequency-domain signal (including frequency, amplitude and phase), which is easy to analyze.

In the program, the analog signal is converted into a digital signal through Analog-to-Digital Converter (ADC) sampling for the sake of the FFT transformation. The MATLAB function fft(x) is utilized to calculate the amplitude of FFT, which is

(4) X=abs(fft(x))

In the formula, x is input digital signal sequence; X is the relative amplitude of the corresponding frequency of x.

In order to calculate the continuous digital signal of pavement noise, the discretization is needed. In a set of equally spaced samples, discrete points are used to approximately substitute the signal of finite length. The expression is as follows:

(5) $$X\left[k\right]=\overset{N-1}{\underset{n=0}{\sum }}x\left[n\right]e^{-j2\pi kn/N}$$

In the formula, X[k] is the relative amplitude of N frequency points; x[n] is sampling signal; N is the length of input sequence, that is, the number of sampling points. It can be inferred from Equation (5) that the frequency resolution can be improved by increasing the sampling point or sampling time.

**Figure 3:**
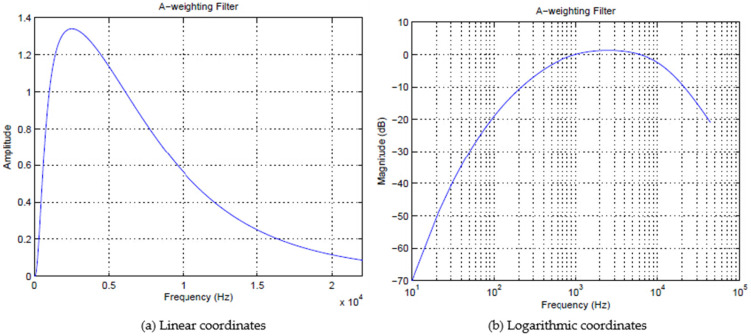
A-weighting filter.

The FFT algorithm can automatically decompose the spectrogram into several discrete frequency regions through the A-weighting filter and then apply the A-weighting to every FFT frequency region, as shown in Figure 3, which simplifies the execution process and increases the frequency resolution.

At first, we should determine the A-weighting filter coefficient α_A_(f) under the frequency of each FFT sample X[k], as shown in Figure 3. The A-weighting FFT sample $X_{A}\left[k\right]$ is given by the following formula:

(6) $$X_{A}\left[k\right]=a_{A}\left(f_{k}\right)X\left[k\right]$$

For the sake of the determination of the A sound level, it is necessary to require the integration of total signal energy. The Parseval’s relation is used to estimate the signal energy in the frequency domain. Parseval’s theorem indicates that the sum (or integral) of the function squares is equal to the sum of the squares of the Fourier transform (or integral), as shown in Equation (7). In addition, since the input signal is the real-value, the samples have complex conjugate symmetry, and the spectrogram is symmetric about half of the frequency samples, as shown in Equation (8).

(7) $$\varepsilon_{x}=\overset{N-1}{\underset{n=0}{\sum }}{\left[x\left[n\right]\right]}^{2}=\frac{1}{N}\overset{N-1}{\underset{k=0}{\sum }}{\left[x\left[k\right]\right]}^{2}$$

(8) $$X\left[\frac{N}{2}+k\right]=X\left[\frac{N}{2}-k\right]$$

Only the signal energy of half of the frequency samples needs to be estimated by utilizing the symmetrical feature of the spectrogram so as to reduce the calculation amount. In order to obtain the final numerical output in dBA form, a reference signal level is also needed. Assuming a suitable reference signal level is ${\tilde{\varepsilon }}_{ref}$, there is:

(9) $$dBA=10\mathrm{lg}\left(\frac{{\tilde{\varepsilon }}_{x}}{{\tilde{\varepsilon }}_{ref}}\right)=10\mathrm{lg}\left({\tilde{\varepsilon }}_{x}\right)-10\mathrm{lg}\left({\tilde{\varepsilon }}_{ref}\right)$$

$10\mathrm{lg}\left({\tilde{\varepsilon }}_{ref}\right)$ is a fixed constant. The signal level expressed in dBA is as follows:

(10) $$N=10\mathrm{lg}\left({\tilde{\varepsilon }}_{ref}\right)+C$$

In the formula, N is the sound pressure level signal level in dBA; C is the calibration constant, which can be determined through laboratory tests.

## 2. Experiment

### 2.1. Material Design

The indoor test mainly uses four types of mixtures: OGFC-13, SMA-13, AC-13 and MS-III at the micro-surface [33]. Both coarse aggregate and machine-made sand use diabase gravel, and the key indicators of aggregate are shown in Table 1. The mineral powder uses limestone ground filler. The aggregate and mineral powder were both supplied by Furong Quarry, Heyuan, Guangdong, China. OGFC-13 uses high-viscosity composite-modified asphalt, SMA-13 and AC-13 use SBS-modified asphalt, and MS-III at the micro-surface uses modified emulsified asphalt. All kinds of asphalt binders were supplied by Guangzhou Xinyue Transportation Technology Co. Ltd., Guangdong, China. The specific indicators are shown in Table 2, Table 3, Table 4, Table 5 and Table 6.

### 2.2. Experiment Procedure

(1) Test condition

To ensure the indoor temperature is 25 °C and the temperature is constant during the test; the tire load is 250 kN; the tire ground pressure is set at 0.7 MPa; the sampling time is 120 s; the microphone is fixed and connected to the computer.

**Figure 4:**
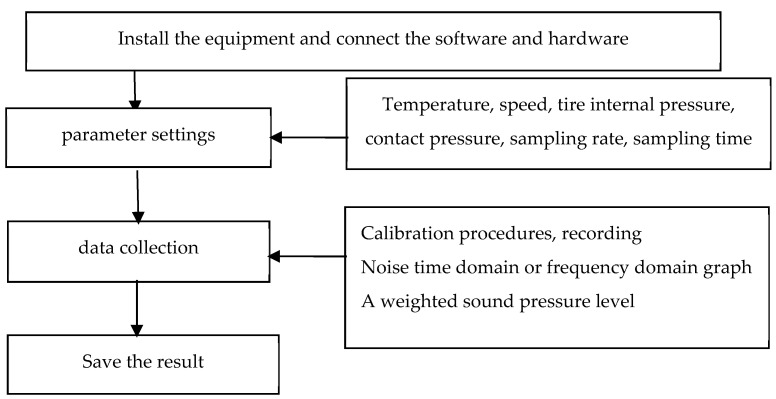
Indoor noise test procedure.

**Figure 5:**
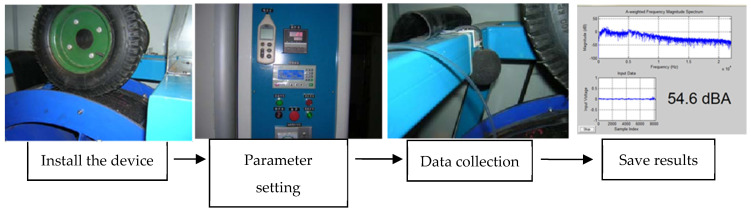
The noise acquisition process.

(2) The noise measurement process is shown in Figure 4 and the noise acquisition process is shown in Figure 5.

(3) Background noise measurement and modification

Before the test, make the driving wheel (that is, the tire) idling without contact with the surface of the test piece. The measured noise at the same position is the background noise, and the value measured in the test is 38.1 dB(A).

In order to ensure the accuracy of pavement noise measurement, it is necessary to determine the background noise of the test system environment [34]. When the difference between the measurement noise and the background noise is more than 10 dB(A), the background noise can be ignored; when the difference is between 6 and 10 dB(A), the measurement noise should be corrected, and the measurement result should be subtracted from the correction value in Table 7; when the difference is less than 6 dB(A), the measurement is invalid.

## 3. Result and Discussion

### 3.1. Impact of Driving Speed on Noise

**Figure 6:**
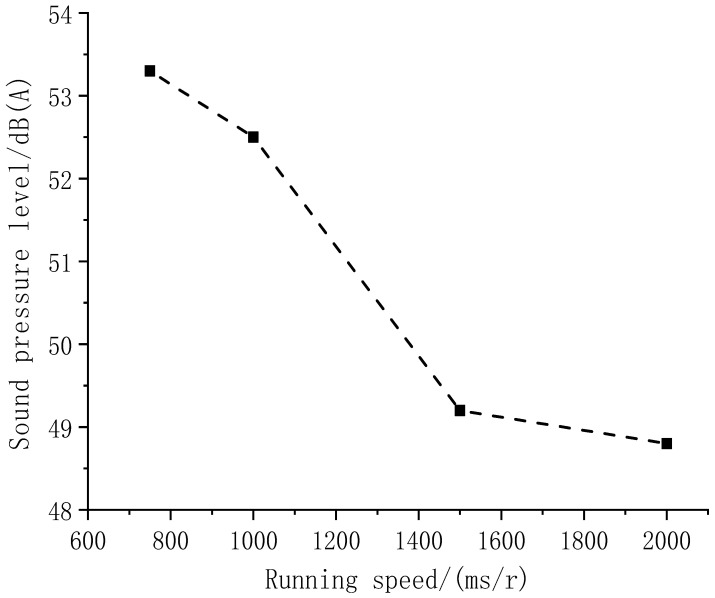
The pavement noise sound pressure level at different driving speeds.

Taking the case of macroporous asphalt pavement, the noise changes of the OGFC pavement are explored at different tire-rolling speeds to adjust the running speed (in terms of the time of one revolution) and record the noise sound pressure level of the OGFC pavement at different running speeds, as shown in Figure 6.

The OGFC–asphalt mixture tire or pavement noise sound pressure level increases with the increment of driving speed, especially at a speed above 1500 ms/r, when this effect is more significant.

**Figure 7:**
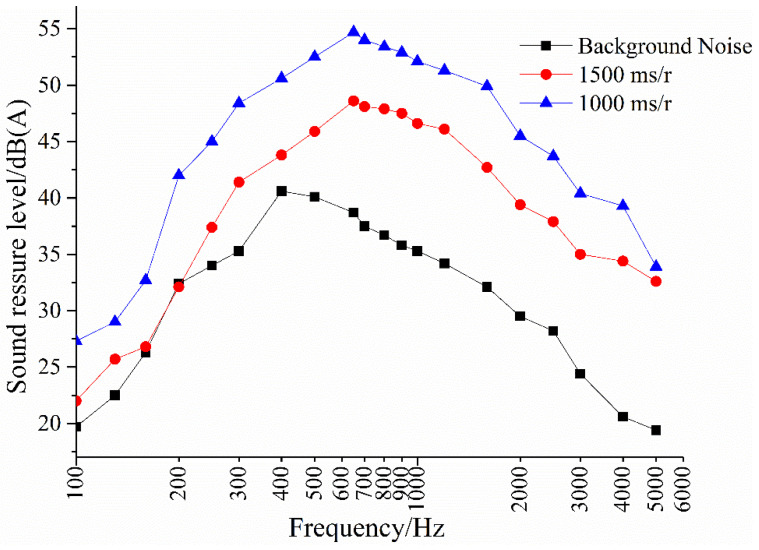
A pavement noise spectrogram at different driving speeds.

A semi-logarithmic spectrum graph is drawn, as shown in Figure 7. It can be seen that OGFC pavement noise is a typical broadband noise, distributed in the frequency range of 200–5000 Hz. The peak value of the spectrum curve at different driving speeds appears in the frequency range of 600–700 Hz. Compared with the background noise, the noise peak value moves to high frequency, which shows that the background environment noise is mainly affected by the driving wheel rotation system, primarily at low frequencies. When the tires interact with the road surface, the pavement noise becomes prominent, and the vibration intensity is greater in the higher frequency range. From the analysis of the frequency spectrum structure, the noise measurement system has a good ability to distinguish pavement noise.

### 3.2. Impact of Wet and Dry Conditions on Noise

This test uses artificial watering to create wet pavement. Water was evenly sprayed on the surface of the test piece to simulate rainfall. The running speed was 1500 ms/r, and the noise test results are shown in Table 8.

**Figure 8:**
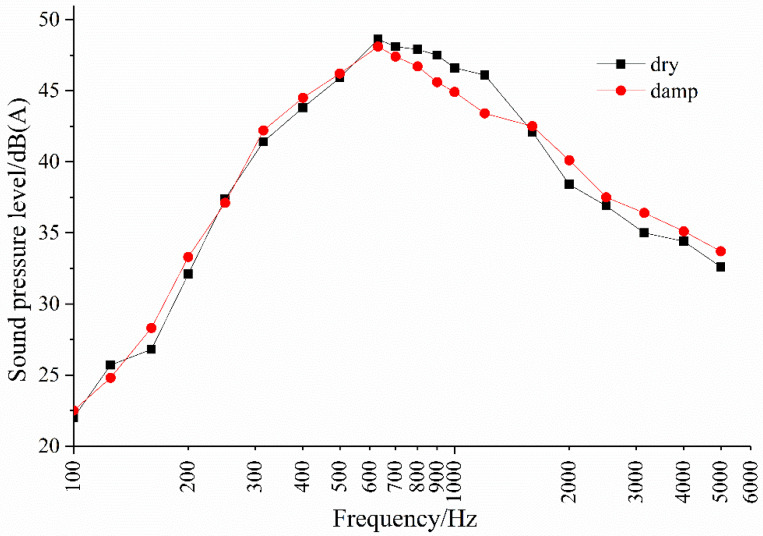
OGFC pavement noise spectrogram under dry or wet conditions.

The test result shows that asphalt pavement is noisier in a wet state than in a dry state. At the same speed, OGFC asphalt pavement with high porosity has a more significant noise reduction effect in a wet state than in a dry state. The frequency spectrum characteristics of OGFC pavement noise under dry and wet conditions are further analyzed in Figure 8.

The low frequency (600 Hz) of OGFC pavement noise under dry or wet conditions does not change significantly. The noise on dry pavement in the mid-frequency (600–1200 Hz) range is slightly greater than that on wet pavement. The increase in sound pressure of wet pavement noise compared to dry pavement noise is mainly reflected in the high-frequency range (≧2000 Hz). The reason is that the water film produced by the OGFC pavement in a wet state reduces the adhesion between the tire and the pavement, causing the noise in the mid-frequency range to decrease, while the amount of water and wheel speed mainly cause the increase in the high-frequency range noise.

### 3.3. Impact of Mixture Type on Pavement Noise

In order to explore the impact of mixture type on pavement noise, four representative pavements, namely porous drainage asphalt pavement (OGFC), ordinary asphalt pavement (AC), asphalt mastic pavement (SMA) and micro-surface pavement (MS-III), are used as the research objects. The measurement test condition is a dry state. The running speed is 1500 ms/r, and the test results are shown in Table 9.

**Figure 9:**
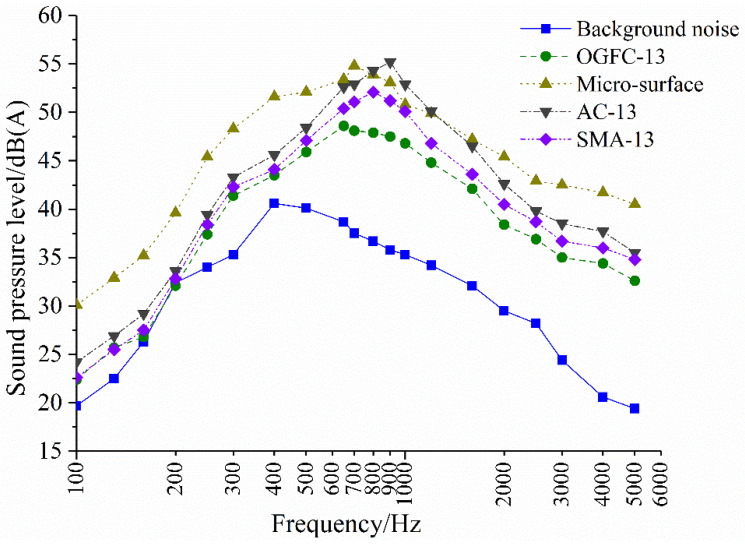
Noise frequency diagram of different pavement types.

It can be known from Table 5 that the porosity and structural depth of OGFC are much larger than those of SMA, AC and micro-surface. The corresponding A-weighted sound pressure level shows that OGFC has the best noise reduction effect, SMA has good noise reduction performance. Meanwhile, AC is poorer, and the micro-surface has the largest noise. The noise mechanism of different pavement structures is not the same, which can be reflected in the composition of the noise. The noise sound pressure level is only a part of the composition. In order to find the main frequency bands that affect the pavement noise, the spectrum analysis of the four pavement noises is performed, as shown in Figure 9.

OGFC, AC, SMA and micro-surface pavements have significant tire or pavement noise characteristics, whose pavement noise spectra are all broad and continuous. The peaks of AC, SMA, micro-surface and OGFC move to low frequencies in sequence. The peak of pavement noise is located in the most sensitive frequency range of the human ears, so this article mainly studies the frequency range of 500–2000 Hz. Comparing the spectrum curve, it is found that the pavement noise difference in the frequency range of 700–1600 Hz is significant, and this frequency domain is the main area where the air pump effect occurs.

There are many pores in OGFC, and the pores are connected to each other and communicate with the outside through the surface. When sound waves occur on the surface of the material, they are reflected or penetrated into the interior and propagate forward, causing the air in the pores to move and create friction with the irregular pore inner walls. The viscous effect and heat conduction effect convert sound energy into heat energy and consume it, which greatly reduces the pumping noise of 700–1600 Hz and effectively reduces pavement noise. The peak frequency is lower than other pavements (600–700 Hz). In previous studies, it was found that the macroporous structure of the pavement changes the propagation characteristics of tire noise, including the fact that the slits in the surface structure directly reduce aerodynamic noise, while the connected pores inside the pavement structure also absorb part of the tire noise [35]. In Europe, there are test results showing that vehicle noise can even be reduced by up to 10 dB(A) with porous pavement structures. European test results show that vehicle noise can even be reduced by up to 10 dB(A) with macroporous pavement structure [36].

Although the porosity of SMA is low, its surface has more coarse aggregates and rich surface texture. With the decrease in the wavelength of the texture structure and the increase in the amplitude, the Helmholtz resonance phenomenon occurs with the reflection in the internal cavity, which provides a free channel for air movement in the contact area and effectively weakens the pumping noise. Compared with AC and micro-surface, SMA pavement noise is also reduced in low-frequency bands, indicating that its dynamic modulus and internal damping help to attenuate the low-frequency noise of tire vibration. The sound pressure level in the full frequency range of the spectrum curve at the micro-surface is far greater than that of other pavements such as AC, especially the low-frequency noise increase rate, which is more obvious. It is because the micro-surface is not compacted, the surface structure is uneven, the top surface of large-size aggregates is uneven, the superposition of common-size aggregates and the fine aggregate enrichment area is recessed, resulting in a significant increase in tire impact noise and vibration noise. Narayanan et al. conducted noise tests on different pavements by orienting the design with different porosity, pore size and morphology of the surface structure (depth, width, shape) and using TPTA (Tire-Pavement Test Apparatus). They initially analyzed that the noise level is related to the depth of the road surface structure, but the influence of the depth of the road surface structure and the porosity inside the pavement remains to be studied [37].

### 3.4. Analysis on the Law of Pavement Noise Attenuation

#### 3.4.1. Impact of Tire Action Times on Noise Decay

It can be known from engineering practice that OGFC has a good pavement noise reduction effect. Nevertheless, with the extension of operating time, the squeeze of the vehicle and the blockage of dust and other debris will result in a smaller porosity and a small increase in the noise level of the OGFC. However, lacking long-term monitoring data of OGFC noise, it is impossible to grasp the law of OGFC noise attenuation.

**Figure 10:**
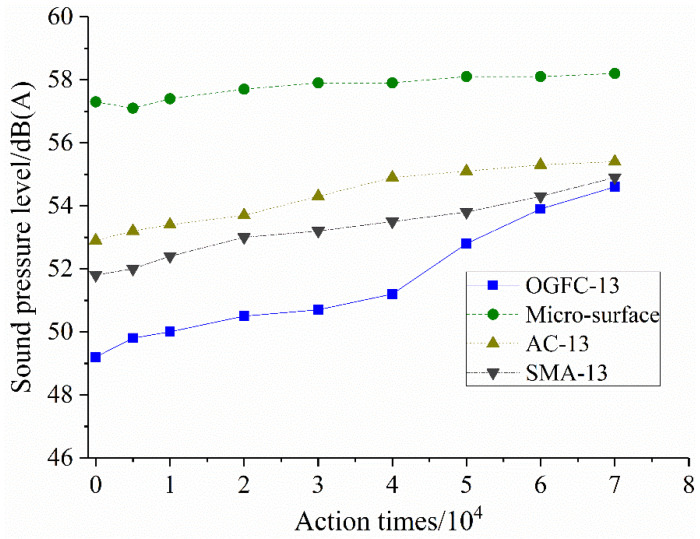
Pavement long-term noise characteristics.

The indoor accelerated loading test system can simulate the impact of long-term traffic load well by increasing the number of tire actions on the OGFC pavement, thereby obtaining the long-term noise characteristics of the OGFC pavement. After the preset number of actions (7 × 104 times), the long-term noise measurement results of OGFC-13, SMA-13, AC-13 and MS-3 at the micro-surface are shown in Figure 10.

It can be seen from Figure 10 that the noise on the four pavements increases with the number of tire actions, among which the tire noise at the micro-surface has always been at the highest level, mainly related to the roughness and flatness of the surface texture. The noise level of densely graded SMA-13 is slightly better than that of AC-13 pavement, which is mainly affected by the damping coefficient of SMA pavement and the larger macroscopic structure depth. The noise attenuation laws of the two densely graded pavements are also similar. As for the large-pore OGFC pavement, its initial noise reduction performance is relatively good, which is at the lowest noise level; when the number of driving operations reaches more than 40,000, the tire noise increases rapidly, which is mainly due to the falling off of aggregate and mortar on the pavement and the decrease in interconnected pores in the mixture. The attenuated noise level in the later stage is closer to the densely graded noise, indicating that the noise reduction function of the macroporous pavement is basically lost at this time. Through the road noise tests at different ages, it was found that the tire noise increased with the aggravation of road wearing [38]. It is consistent with our main finding of the four pavement noise variation patterns under the actions of different wheel-tires conducted by the laboratory accelerated loading system developed in this paper. This also proves that the device can simulate the action of wheel tires on the pavement well.

**Figure 11:**
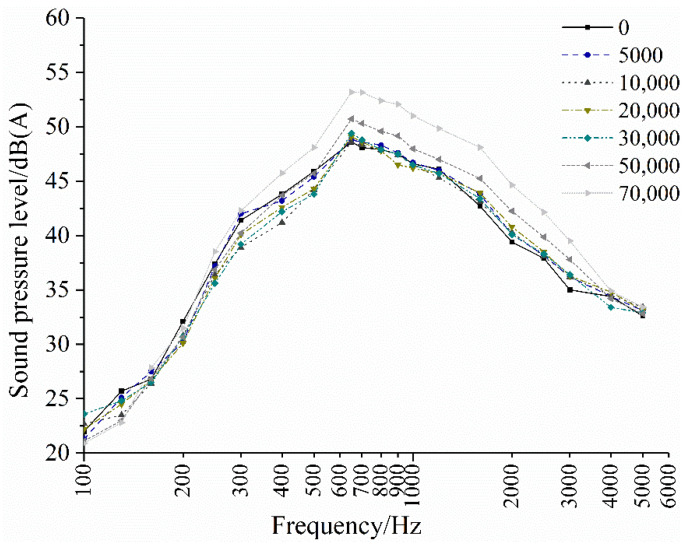
Changes in the OGFC pavement noise characteristics.

According to the frequency spectrum analysis based on the measured noise data, the changes in the noise characteristics of OGFC are observed as the number of tire actions increases, as shown in Figure 11. The noise level of the newly built OGFC pavement is greater than that of the OGFC pavement after the action of tires in the range of 100–600 Hz, while the higher-frequency domain above 700 Hz is the opposite, which is because the effect of the traffic load may cause the single stone protruding on the OGFC pavement to fall off and become smoother, the impact on the tires is reduced, and the low-frequency vibration and noise generated are also reduced accordingly. Furthermore, with the compaction of the tires, the connected porosity of the OGFC pavement decreases, and the effect of reducing the pumping noise becomes smaller accordingly. The internal and external pressure between the tire and the road cannot be kept constant, resulting in increased noise in the high-frequency range of the OGFC pavement, and the peak of the spectrum has to move to the high-frequency direction.

#### 3.4.2. Impact of Structural Depth on Noise Decay

**Figure 12:**
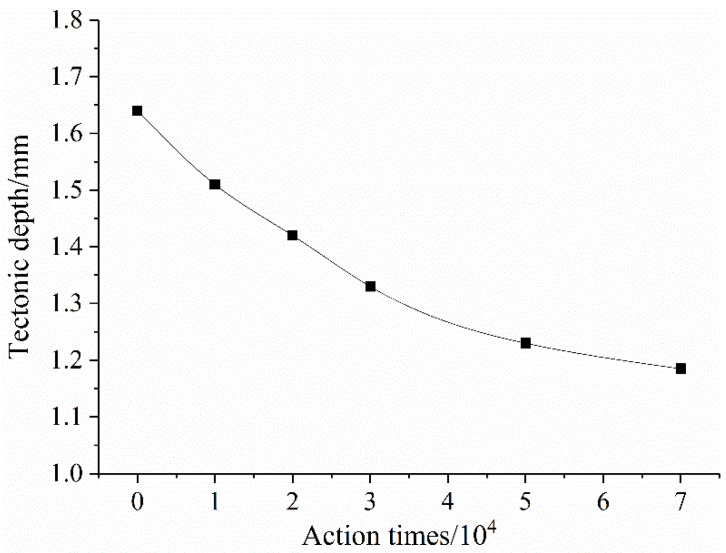
OGFC structural depth change curve.

After OGFC pavement has passed tire action times (7 × 10^4^ times), the structural depth change is shown in Figure 12. OGFC pavement is compacted under the action of tires, and the structural depth decreases rapidly, and then the decrease rate becomes slower and tends to be stable.

**Figure 13:**
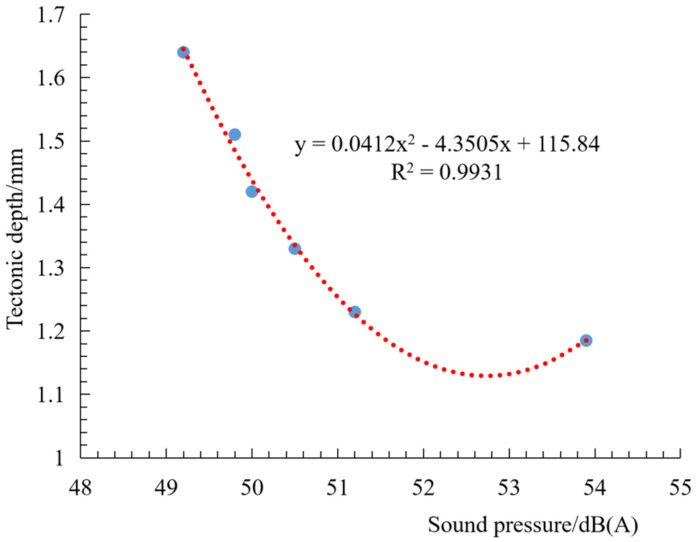
The relationship between structural depth and noise.

The correlation between the structural depth attenuation process and noise of constructing a large-pore pavement is shown in Figure 13. OGFC structural depth and noise show a good secondary correlation. As the number of tire actions increases, the structure depth decreases, OGFC pavement noise tends to increase, and the correlation is good. The main reason is that the initial OGFC pavement has a large structural depth, rich surface texture, and dense through-holes formed on the surface and inside of the road, which has good sound absorption performance. When the depth of the structure becomes lower under the load of the tire, the connected pores are correspondingly reduced, and the air in the pores is squeezed when the tire interacts with the road surface. When the interaction between the tire and the road surface ends, the pores suck in a large amount of air due to the imbalance of internal and external pressure, and the pumping noise increases.

## 4. Conclusions

(1) OGFC tire or pavement noise increases with the increase in driving speed, and the noise structure is different at different speeds. OGFC pavement noise at low speeds increases the noise level in the high-frequency range due to tire or road sticky action. Regarding the noise at higher speeds, in addition to the effect of sticky action, there is also pumping noise and vibration noise from tire patterns, which violently beat the pavement.
(2) The noise reduction performance of OGFC comes from its porosity up to 20%, which effectively reduces air pump noise. SMA has a rich surface texture and high internal damping, and its noise reduction performance is inferior only to OGFC pavement. The higher noise at the micro-surface is due to the unevenness and nonuniformity of the pavement macroscopic structure. The noise reduction effect of different asphalt pavements is OGFC-13> SMA-13> AC-13> MS-III.
(3) With the decrease in the structural depth, the OGFC pavement noise has an increasing tendency, and the OGFC pavement structural depth has a good quadratic parabolic relationship with the noise sound pressure level. In the later stage of the driving action, the noise reduction function of OGFC tends to disappear due to the speed of the connected pores. Meanwhile, the pavement noise characteristics are similar to those of densely graded asphalt pavement.

Future studies will be focused on the slipperiness characteristics and the in situ validation of the macroporous asphalt pavement based on the weighted sound pressure level sensor.

## Figures and Tables

**Table 1:** Technical indicators of coarse aggregate.

Test Item	Technical Requirement		Test Result	Monomial Assessment
	Unit	Design Requirement		
Stone crushing value	%	≤15	7.9	Eligibility
Los Angeles abrasion loss	%	≤22	9.4	Eligibility
Apparent relative density	—	≥2.60	2.888	Eligibility
Adhesion to modified asphalt	Level	≥5	5	Eligibility
Polish value	—	≥42	45	Eligibility

**Table 2:** The technical index of fine aggregate.

Sample Specifications			0–3 mm	
Test Item	Technical Requirement		Test Result	Monomial Assessment
	Unit	Design Requirement		
Apparent relative density	—	≥2.50	2.909	Eligibility
Robustness (>0.3 mm part)	%	≤12	2.5	—
Sand equivalent	%	≥65	73	Eligibility
Angularity (flow time)	s	≥30	39.6	Eligibility

**Table 3:** The technical index of high-viscosity modified asphalt.

Test Item		Technical Requirement	Test Result	Monomial Assessment
Penetration 25 °C, 100 g, 5 s, 0.1 mm		40	49	Eligibility
Ductility 5 °C, 5 cm/min, cm		≥50	70	Eligibility
Softening point (°C)		≥80	>90	Eligibility
Flash point (°C)		≥260	337	Eligibility
Viscosity 25 °C (N*m)		≥25	28	Eligibility
Tenacity 25 °C (N*m)		≥15	16	Eligibility
60 °C dynamic viscosity (Pa.S)		>250,000	>580,000	Eligibility
Rolling thin film oven test (RTFOT) Residue (163 °C, 85 min)	Mass change (%)	±1.0	−0.054	Eligibility
	Penetration ratio (%)	≥65	79.5	Eligibility

**Table 4:** The technical index of SBS-modified asphalt.

Test Item		Technical Requirement	Test Result	Monomial Assessment
Penetration 25 °C, 100 g, 5 s, 0.1 mm		40–60	54	Eligibility
Penetration index PI		≥+0.0	+0.17	Eligibility
Ductility 5 °C, 5 cm/min, cm		≥20	34	Eligibility
Softening point (°C)		≥75	86.5	Eligibility
Flash point (°C)		≥230	340	Eligibility
Solubility (%)		≥99	99.8	Eligibility
Storage stability *: 163 °C, 48 h, poor softening point °C		≤2.0	1.2	Eligibility
Elastic recovery 25 °C, %		≥90	96	Eligibility
Kinematic viscosity (Pa·s)	135 °C	≤3	2.38	Eligibility
	165 °C	No requirement	0.62	
Rolling thin film oven test (RTFOT) Residue (163 °C, 85 min)	Mass change (%)	±1.0	−0.016	Eligibility
	Ductility 5 °C, 5 cm/min, cm	≥20	21	Eligibility
	Penetration ratio (%)	≥65	81.6	Eligibility

**Table 5:** The technical index of modified emulsified asphalt.

Test Item		Unit	Technical Requirement	Test Result	Monomial Assessment
Residue on sieve (1.18 mm) sieve, no more than		%	≤0.05	0.03	Eligibility
Particle charge		—	positive ion (+)	positive ion	Eligibility
Viscosity (Asphalt Standard Viscometer C_25,3_)		s	8–25	18	Eligibility
Evaporation residue	Residual content	%	≥63	65.3	Eligibility
	Penetration (25 °C)	0.1 mm	40–150	79	Eligibility
	Softening point	°C	≥55	66.0	Eligibility
	Ductility (5 °C)	cm	≥25	51	Eligibility
	Solubility	%	≥97.5	99.7	Eligibility
	Elastic recovery (10 °C)	%	≥60	71	Eligibility
Storage stability (1 d)		%	≤1	0.5	Eligibility

**Table 6:** The mixture design index.

Sieve Mesh	16	13.2	9.5	4.75	2.36	1.18	0.6	0.3	0.15	0.075	Asphalt-Aggregate Ratio (%)	Porosity (%)
OGFC-13	100	95	55.9	11.8	11.4	11	8.4	7	5.5	4.2	5.2	21.6
AC-13	100	90	68	38	24	15	10	7	5	4	4.3	3.8
SMA-13	100	89	63	25	19	15	14	13	12	10	5.9	4.1
MS-III	100	100	100	90	70	50	34	25	18	15	7.2	3.8

**Table 7:** Background noise correction value.

Difference Between the Measurement Noise and the Background Noise/dB(A)	6–8	9–10	>10
Correction value	1	0.5	0

**Table 8:** A comparison of OGFC pavement noise in dry and wet conditions.

Pavement State	Sound Pressure Level/dB(A)	Noise Reduction Level/dB(A)
Dry	49.2	0.6
Wet	49.8	

**Table 9:** Measurement and analysis results of different pavement noises.

Gradation Type	Background Noise	OGFC-13	MS-III	AC-13	SMA-13
Porosity /%	—	21.6	3.8	3.8	4.1
Structural depth/mm	—	1.94	0.83	0.42	1.14
Sound pressure level /dB(A)	38.1	49.2	57.3	52.9	51.8

## Data Availability

The data presented in this study are available on request from the corresponding author.
